# Effectiveness and Safety of Immune Checkpoint Inhibitors for Patients with Advanced Non Small-Cell Lung Cancer in Real-World: Review and Meta-Analysis

**DOI:** 10.3390/cancers13061388

**Published:** 2021-03-19

**Authors:** Manlio Mencoboni, Marcello Ceppi, Marco Bruzzone, Paola Taveggia, Alessia Cavo, Francesca Scordamaglia, Marina Gualco, Rosa Angela Filiberti

**Affiliations:** 1 SSD Oncologia Ospedale Villa Scassi, ASL 3 Genovese, 16149 Genova, Italy; manlio.mencoboni@fastwebnet.it (M.M.); Paola.taveggia@asl3.liguria.it (P.T.); Alessia.cavo@asl3.liguria.it (A.C.); 2 Clinical Epidemiology, IRCCS Ospedale Policlinico San Martino, 16132 Genova, Italy; marcello.ceppi@hsanmartino.it (M.C.); marco.bruzzone@hsanmartino.it (M.B.); 3 SC Pneumologia Ospedale Villa Scassi, ASL 3 Genovese, 16149 Genova, Italy; Francesca.scordamaglia@asl3.liguria.it; 4 SC Anatomia Patologica Ospedale Villa Scassi, ASL 3 Genovese, 16149 Genova, Italy; marina.gualco@asl3.liguria.it

**Keywords:** non-small cell lung cancer, NSCLC, immunotherapy, immune checkpoint inhibitors, nivolumab, pembrolizumab, atezolizumab, real-life, efficacy, safety

## Abstract

**Simple Summary:**

The benefit of programmed death-1/programmed death ligand-1 (PD-1/PD-L1) immunotherapy, particularly of nivolumab, pembrolizumab and atezolizumab, in the second-line setting of patients with non-small cell lung cancer has been documented in randomized clinical trials, showing improvements in global survival and in the overall response rate. Nevertheless, patients enrolled in these studies met strict eligibility criteria, allowing for the treatment of patients that do not reflect the broader oncology patient population. Experiences from real-world data are useful in providing further evidence of the benefit of treatment in a wider range of patients, including those underrepresented in clinical trials. We performed a meta-analysis to evaluate the outcomes in non-small cell lung cancer patients treated in everyday practice with these drugs as the second line, and more generally with immunotherapy with checkpoint inhibitors (ICIs), showing that the efficacy and safety were comparable to those in selected studies. Results may encourage to treat patients excluded from randomized studies.

**Abstract:**

Immunotherapy based on anti PD-1/PD-L1 inhibitors is the new standard of advanced non-small cell lung cancers. Pembrolizumab, nivolumab and atezolizumab are used in clinical practice. The strict eligibility criteria of clinical trials do not allow researchers to fully represent treatment effects in the patients that will ultimately use these drugs. We performed a systematic review and a meta-analysis to evaluate the effectiveness and safety of these drugs, and more generally of ICIs, as second-line therapy in NSCLC patients in real world practice. MEDLINE, PubMed, Scopus and Web of Science were searched to include original studies published between January 2015 and April 2020. A total of 32 studies was included in the meta-analysis. The overall radiological response rate (ORR), disease control rate (DCR), median progression-free survival (PFS) and overall survival (OS) were 21%, 52%, 3.35 months and 9.98 months, respectively. The results did not change when analysis was adjusted for Eastern Cooperative Oncology Group performance status (ECOG PS) and age. A unitary increase in the percent of patients with liver and CNS metastases reduced the occurrence of DCR by 7% (*p* < 0.001) and the median PFS by 2% (*p* = 0.010), respectively. The meta-analysis showed that the efficacy and safety of immunotherapy in everyday practice is comparable to that in clinical trials.

## 1. Introduction

Immunotherapy with checkpoint inhibitors (ICIs) that target the PD-1/PD-L1 (programmed death-1/programmed death ligand-1) pathway is considered the new standard treatment of advanced and metastatic squamous and non-squamous lung cancers, with or without chemotherapy [1,2,3,4,5,6,7,8,9]. Nevertheless, cancer patients enrolled in randomized clinical trials (RCT) meet strict eligibility criteria allowing for the treatment of patients that do not reflect the more heterogeneous population of oncologic patients. Being nonselective, the reported experience of immunotherapy described in real-world studies may provide complementary data to clinical trial findings [10,11].

Experiences from real-world data have been published, suggesting that the efficacy and safety profiles of immunotherapy are consistent with those reported in clinical trials [10,12]. These studies are useful in providing further evidence of the benefits of treatments in a wider range of patients, including those underrepresented in clinical trials [13,14,15].

Anti-PD-1 antibodies, such as pembrolizumab and nivolumab, and anti-PD-L1 antibodies such as atezolizumab have been approved as a second-line therapy, and are now being used in clinical practice. We reviewed the available literature and performed a meta-analysis with the aim of describing the benefits and risks of these drugs, and more generally of ICIs, as the second-line therapy in the management of non-small cell lung cancer (NSCLC) patients in real practice. More specifically, we evaluated the activity of ICIs in terms of response, progression-free survival (PFS) and overall survival (OS). By means of the meta-analysis, we estimated the pooled means of ORR (objective response rate), DCR (disease control rate), PFS and OS as reported in the non-randomized studies, and compared them with the analogous values estimated from clinical trials. Since we only considered observational studies in which there were no strict criteria for patient inclusion or randomization, we considered it appropriate to show the results adjusted by age and health status of the patients.

## 2. Methods

### 2.1. Search Strategy and Inclusion Criteria

A literature search was performed using the MEDLINE, PubMed, Scopus and Web of Science databases to include studies published in English between January 2015 and April 2020. The following terms were used: “(Non-small cell lung cancer OR NSCLC) AND (nivolumab OR atezolizumab OR pembrolizumab OR immune checkpoint inhibitor OR PD-1 OR PD-L1 OR “programmed cell death 1” OR “programmed cell death ligand 1”) AND (real-life OR real-world OR real routine OR retrospective OR everyday practice OR daily practice OR clinical practice). The reference lists of eligible articles were searched for additional studies.

### 2.2. Eligibility Criteria

To be eligible, studies needed to have accrued patients treated in the second and further lines of therapy, and to provide data available for tumor response and/or survival analysis. 

Reviews without original data, meta-analyses, clinical trials, conference abstracts, unpublished studies and case reports were excluded. Studies on selected cases (focusing on gene mutations or specific diseases or systemic therapies) or those enrolling fewer than 50 patients were also excluded. The complete manuscript of all relevant studies was retrieved. 

### 2.3. Data Extraction

Data were extracted by one investigator and confirmed by another (R.A.F., M.B.) according to a predefined data extraction form. Disagreements were resolved by consensus. 

The extracted data included first author, publication year, number of patients, study drug, participant and tumor characteristics (sex, age, smoking status, tumor stage, histology, Eastern Cooperative Oncology Group performance status (PS), prior radiotherapy, CNS and liver metastases, line of treatment and median follow-up time). The reported clinical outcomes were listed according to the RECIST criteria as complete response rate (CR), partial response rate (PR), stable disease rate (SD), ongoing/objective response rate (ORR, as the sum of CR and PR), disease control rate (DCR, as the sum of stable disease rate and ORR), median progression-free survival (PFS, months) and median overall survival (OS, months). The rates of drug adverse events (AEs) and immune-related adverse events (irAEs), of any-grade and grade 3–4, were also extracted. 

The endpoints of the pooled analyses were ORR, DCR, PFS and OS. Only studies reporting confidence intervals were included in PFS and OS analysis.

## 3. Statistical Analysis

In our analyses we considered as effect size the proportion of ORR and DCR and median PFS and OS. The meta-analysis was carried out by fitting the random effects model, as suggested by DerSimonian and Laird [16]. 

This model allowed us to estimate the amount of variability between studies, and accordingly provided suitable estimates of the standard errors of the parameters.

Due to the heterogeneous healthy status of the patients enrolled in the studies, each pooled estimate was also adjusted by the performance status index (PS), or by the presence of CNS or liver metastases by means of the meta-regression model [17]. Pooled estimates were also adjusted by age.

To verify if some studies strongly influenced the pooled estimates, a sensitivity analysis (excluding studies one at a time and estimating the parameters again) was performed. Concerning publication bias, we explored the possibility that only selected studies were published by applying Egger’s test [18]. The analysis was carried out using the procedure METAN to fit the random effects model, and the diagnostics procedures METAINF and METABIAS developed by STATA software (StataCorp. 2015. Stata: Release 14.2. Statistical Sofware. College Station, TX, USA: StataCorp LP.).

## 4. Results

### 4.1. Study Characteristics

**Figure 1:**
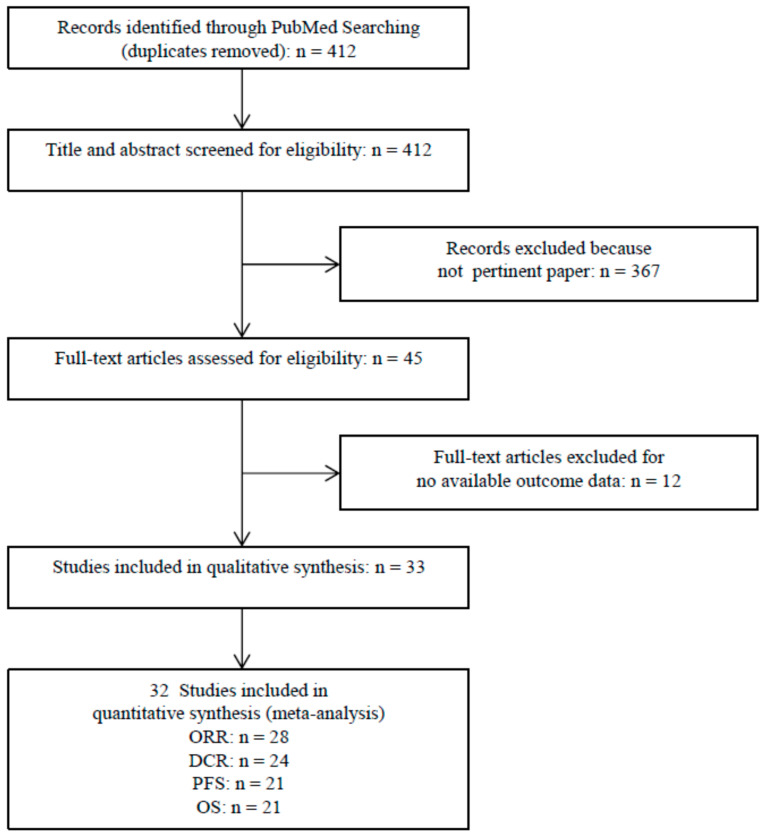
Flow chart of study selection and design.

After excluding duplicates, the first literature search yielded a total of 412 citations. Following a review of the titles and abstracts, the full texts of 45 potentially eligible studies were analyzed. Of these, 33 met the inclusion criteria for the qualitative synthesis [19,20,21,22,23,24,25,26,27,28,29,30,31,32,33,34,35,36,37,38,39,40,41,42,43,44,45,46,47,48,49,50,51], while 32 studies fulfilled the eligibility criteria for inclusion in the meta-analysis [19,20,21,22,23,24,25,26,27,28,29,30,31,32,33,34,35,36,37,38,39,40,41,42,43,44,45,46,47,48,49,51]. Figure 1 shows the selection process.

Table 1 summarizes the baseline characteristics of the 33 studies evaluating single or combination therapies with nivolumab (31 studies), pembrolizumab or atezolizumab, or more generally with immune checkpoint inhibitors. The sample size of each study ranged from 50 to 2071, with four studies exceeding 1000 patients [30,33,38,40].

Most of the studies included patients with mixed histological types, while two studies enrolled only squamous or non-squamous tumors [31,33]. The patients were males in 43 to 84% of cases, and they had a median age ranging from 58 to 75 years in 27 studies. The percentage of smokers was 54 to 92% in 30 of the studies. The proportion of patients with PS ≥ 2 ranged from 4 to 49% in 32 studies. Only 22 and 12 studies reported on the presence of CNS or liver metastases, respectively. On average, the second-line ICIs treatment was given to 48% of patients (range 16–100%). 

Table 2 summarizes the patients’ outcomes. The median follow-up length as reported in 18 studies ranged from 4.9 months to 26.6 months.

Twenty-eight studies provided data for ORR. The rates ranged from 7 [28] to 36% [26,36]. Twenty-four studies provided data for DCR, which was achieved in 30 [37] to 67% of cases [26].

The median PFS was reported in 28 studies and ranged from about 2 months to 7 months [26].

Median OS was reported in twenty-six studies and ranged from 5.8 months in a study with 175 patients [42] to 18 months in two studies with a smaller sizes (97 and 70 patients, respectively) [20,34]. 

Overall, noticeable efficacy outcomes greater than 30% for ORR and 65% for DCR were observed in two recent series of patients, with good performance status, PS 0-1, in 96% of them [25,26]. An OS of 13 months was reported in only one of these studies [26]. 

Among studies reporting survival data, a better prognosis with median OS ranging from 13 to 18 months was observed in seven reports [20,22,26,27,29,34,46]. Among these, 75 to 96% of patients had PS 0-1 and median age from 58 to 69 years. CNS metastases were reported in 9 to 44% of patients in three out of the seven studies [27,29,34]. No differences with respect to all other studies were observed in the three analyses with the majority of patients aged more than 70 years [19,36]. 

Toxic effects were reported in twenty-six studies: total any-grade AEs occurred in 29 to 88% of patients in 16 studies. Overall, grade 3 or higher EAs were reported in 5 to 28% of cases in 14 studies. Nine studies showed irAEs, which were observed in 7 to 46% of patients and in 3 to 11% of patients for grade 3 and 4 irAEs, respectively.

A number of papers reported on the role of age, tumor histology, PS, and smoking status. Overall, age did not appear to be an independent predictor of efficacy [23,33,35,40,41,44,45,48]. Furthermore, the incidences of AEs of all grades and of grade 3–4 were also similar between the age groups according to some papers [23,33].

One out of the six authors analyzing histology found a positive association between squamous tumors and patients’ prognosis or response [51], and one found an OS superiority for the non-squamous type [35], but the other authors did not find differences between the histotypes [29,40,41,48]. 

In papers addressing PS, an adverse effect on OS was observed for patients with an ECOG PS above 0 [31,41,48] or 1 [22,23,29,33,35,36,40,42,44,49]. A halved OS for PS 2 vs. PS 0–1 patients was found by Juergens et al. [44] and, on average, patients with PS 2 or above lived seven months less than patients with a better status [26,35,36,41,42,44,48].

An ECOG score greater than 2 was also associated with a shorter time to recurrence [23,35,36,43,47,49]. In addition, patients with PS 2 experienced a disease control rate of 26%, compared to 50% in patients with better status [43]. However, other authors found no significant differences with regard to PFS between the subgroups [44]. Safety outcomes seemed not to be affected by PS [43]. 

An association between the presence of SCN metastases and a poor prognosis was evidenced [41,44], but no measurable impact on OS was observed in a larger number of papers [29,33,48]. 

Liver metastases were correlated with a lower OS in five papers [30,31,33,48,49], and with a significantly shorter progression-free survival in one paper [47]. 

Smoking was significantly and independently associated with a more favorable PFS in one study [43], but did not appear to influence survival according to other authors [48].

Finally, the occurrence of irAEs was associated with better outcomes in terms of OS, PFS and response rate in the paper of Baldini et al. [30], and of PFS in the paper of Nakaya [46].

### 4.2. Meta-Analysis

#### Pooled Analysis of ORR and DCR

In total, 28 and 24 studies were available for ORR and DCR analysis, reporting on 8312 and 7447 patients, respectively. The non-adjusted pooled estimate for ORR was 21% (95%CI: 19–23%), and for DCR it was 52% (95%CI: 48–55%).

**Figure 2:**
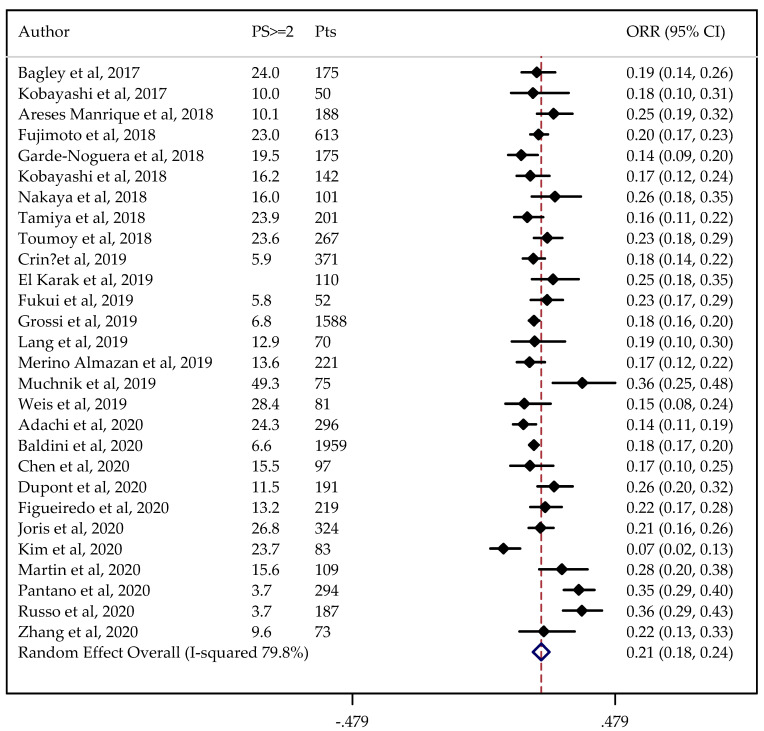
Forest plot of non-small cell lung cancer (NSCLC) objective response rate (ORR) adjusted for performance status (PS). The ORRs were either taken directly from individual studies or calculated using reported numbers of responding and total treated patients. PS ≥ 2: proportion of patients with PS ≥ 2.

**Figure 3:**
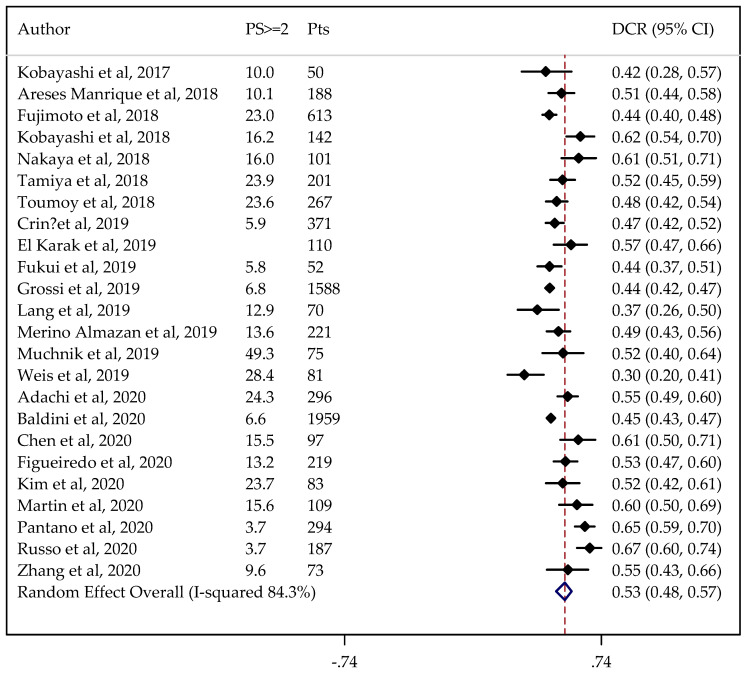
Forest plot of NSCLC disease control rate (DCR) adjusted for performance status (PS). The DCRs were either taken directly from individual studies or calculated using reported numbers of responding, stable, and total treated patients. PS ≥ 2: proportion of patients with PS ≥ 2.

Information on PS status was not available in one case. The results did not change when the analysis was adjusted for PS, considered to be predictive of the outcomes under study (ORR = 21%, 95%CI: 18–24% and DCR = 53%, 95%CI: 48–57%) (Figure 2 and Figure 3). 

The estimated percentage of ORR and DCR adjusted for PS did not change after removing from the analyses the studies with major differences. In the sensitivity analysis, the pooled ORR and DCR ranged from 21 to 22% and from 51 to 54%, respectively.

Table 3 shows the results obtained when adjusting for age and presence of CNS or liver metastases. In this case, a slightly lower DCR was found for patients with liver metastases (0.47, 95%CI: 0.46–0.49). 

An assessment of the effects of confounders on our endpoints showed no significant effects for age, PS, and CNS metastases, while a unitary increase in the percent of patients with liver metastases reduced the occurrence of DCR by 7% (*p* < 0.001) (Table S1). 

### 4.3. Pooled Analysis of PFS and OS

Pooled analyses of PFS and OS were performed on studies including measures of uncertainty. 

The median PFS was analyzed in 21 studies involving 7256 patients. The median OS was evaluated in 21 cases comprising 10,707 patients.

The non-adjusted pooled median for PFS was 3.35 months (95%CI: 3.05–3.68 months), and for OS was 9.98 months (95%CI: 9.17–10.86 months).

**Figure 4:**
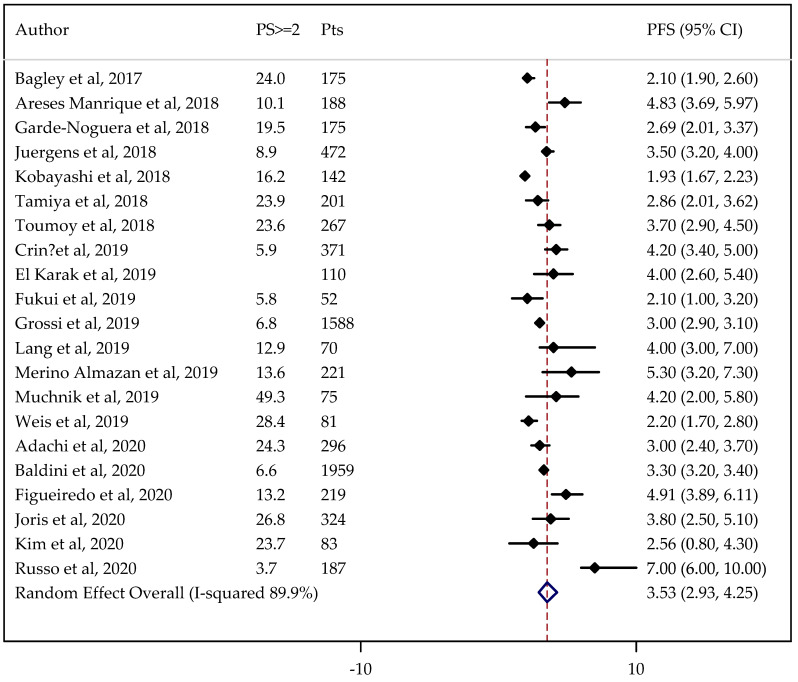
Forest plot of NSCLC progression-free survival (PFS) adjusted for performance status (PS). PS ≥ 2: proportion of patients with PS ≥ 2.

**Figure 5:**
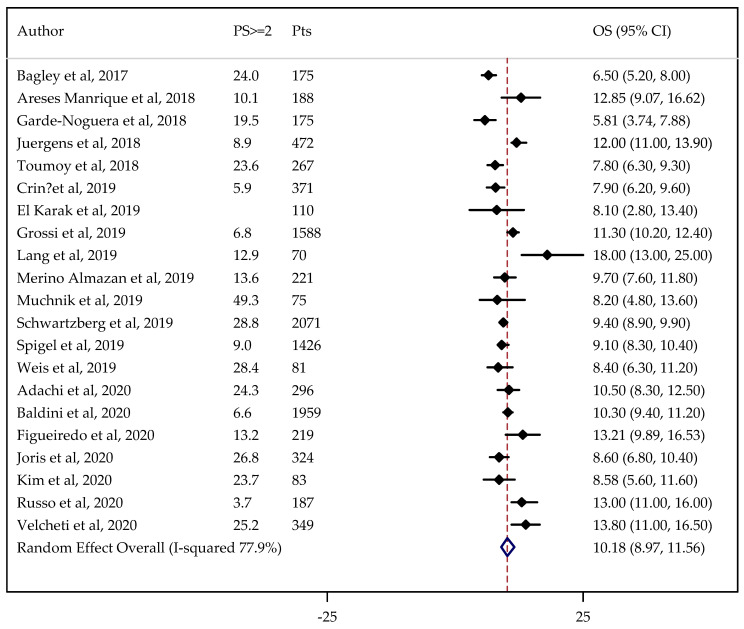
Forest plot of NSCLC overall survival (OS) adjusted for performance status (PS). PS ≥ 2: proportion of patients with PS ≥ 2.

Information on PS status was not available in one case. The results did not change after adjustment by PS being 3.53 months (95% CI: 2.93–4.25 months) for PFS (Figure 4) and 10.18 months (95%CI: 8.97–11.56 months) for OS (Figure 5). 

The estimated median PFS and OS did not change after removing from analysis the studies with major differences. In the sensitivity analysis, the pooled PFS and OS ranged from 3.29 to 3.66 months and from 9.93 to 10.45 months, respectively.

Adjusting for age and the presence of metastases, a slightly lower OS was found for patients with liver metastases (8.65, 95%CI: 7.23–10.36; Table 3). 

When assessing the role of confounders, median PFS was reduced by 2% along with the unitary increase in the percent of patients with CNS metastases (*p* = 0.014) (Table S1).

The results of statistical tests indicated considerable heterogeneity (*I*^2 ^ >  75%) for all evaluated outcomes. 

Taking into account that we considered only studies involving at least fifty patients, the results from the Egger test (*p* = 0.503 for ORR and *p* = 0.962 for OS) suggested that publication bias was not an influential factor.

## 5. Discussion

In this review and meta-analysis we evaluated the benefits of immune checkpoint inhibitors, especially the PD-1/PD-L1 inhibitors nivolumab, pembrolizumab and atezolizumab, as a second-line therapy in the management of advanced or metastatic NSCLC in real-world clinical practice. 

Immunotherapy is currently recommended as the treatment of choice for patients undergoing disease progression after third-line therapy [1,2,3]. Although some studies have shown that the benefits seem to be superior for specific histotypes [52], or are only seen in ever-smokers [53,54], there is also evidence of an improved efficacy and tolerability of second-line immunotherapy with respect to standard chemotherapy in patients with different sites of metastasis, chemotherapy combinations (independent of tumor PD-L1 expression), and histology [54]. Promising results have been achieved in all age groups, although the clinical benefit was less certain for patients older than 75 years [1,5,55]. 

Some authors found no benefit with nivolumab in terms of OS, PFS, or overall response rate, but for a lower percentage of grades 3 or 4 AEs [56], nivolumab has been associated with significantly longer overall survival, and has a good safety profile in both squamous [5] and non-squamous NSCLC [7]. Compared with other PD-1 inhibitors (pembrolizumab and atezolizumab), nivolumab has been estimated as the best option in terms of anti-tumor efficacy, while atezolizumab is better at reducing adverse events, and pembrolizumab monotherapy was shown to be the best especially for patients with PD-L1 ≥ 50% [57].

Patients participating in RCTs are generally younger and healthier than patients in the general population, are more likely to tolerate treatment, derive clinical benefits, and have a better performance status [10]. The strict eligibility criteria do not allow RCTs to fully represent the treatment effects in the patients that will ultimately use the drugs. 

In real-world settings, patients are more heterogeneous. Generally, studies in clinical practice evaluate a meaningful proportion of patients not enrolled in clinical trials, including 30 to 50% of patients with advanced age, ECOG performance status ≥2, multiple co-morbidities, and/or brain metastases [10]. This way, real-world data may be helpful to evaluate the reproducibility of outcomes of novel treatments, and to identify new information on efficacy that can be used in treatment decision-making [10,11]. 

Analyses on the use of immunotherapy in NSCLC have already been published, reporting endpoints, such as overall survival and the presence of AEs, that are generally consistent with each other and with the outcomes observed in RCTs [11,12,58,59]. 

Following the selection criteria, 33 studies entered our prior qualitative analysis, reporting a median overall survival ranging from 5.8 to 18 months. In accordance with the literature, most of the studies that analyzed the role of age showed that older patients with good performance status may derive the same benefits as younger subjects in terms of OS, PFS or tolerability [12,23,31,33,35,41,43,44,45,48,60]. 

Among the RCTs studies including patients with PS 2 according to ECOG classification, the same tolerability but a lower efficacy outcome was found when compared to patients with better PS [40,55].

In our selected real-world studies, patients with PS 2 constituted from 4 to 49% of the series. Most of the papers addressing this population subset found worse survival in ECOG PS 2 patients compared to those with better PS [31,41,42,44], and some analyses have identified PS as the only independent prognostic factor [22]. 

Contrasting results were obtained regarding the prognostic role of histology [35,51], even if the majority of the authors did not find differences between squamous and non-squamous tumors [29,40,41,48]. 

Generally, in accordance with other real-world analyses [12], the studies in this review provided evidence that immunotherapy is also safe and effective in patients with stable CNS metastases [29,31,33,48]. At the same time, five authors found that immunotherapy had no benefit in terms of the survival of patients with liver metastases [30,31,48,49].

Adverse events were reported in almost all the trials that tested immunotherapy [3]. The safety profile in our analysis was generally consistent with the literature on RCTs: total adverse events ranged from 29 to 88% for any grade, and from 5 to 28% for grades 3–4, which is opposed to 36–69% and 6–40%, respectively, with different therapies [5,7,8,9,55,61,62].

### 5.1. Quantitative Analysis

The objective of this meta-analysis was to quantitatively synthesize what is known about the benefits of immune checkpoint inhibitors as used in the real-world clinical management of NSCLC patients, regarding treatment response, tumor progression and survival.

As we included observational studies, and the outcomes we evaluated can be affected by confounding, the pooled analysis was adjusted for performance status, which was assumed to be the most significant predictor of clinical benefit [40,55,59] for age and the presence of CNS or liver metastases.

Under the unadjusted analysis, ORR and DCR were achieved in about 21 and 53% of patients, respectively. The median PFS and OS were about 3 and 10 months, respectively.

Assessment of the effect of confounders on our endpoints showed no significant effects for age and PS, while an increase in the number of patients with liver metastases significantly lowered DCR by 7%. Although liver metastases have a slightly greater PFS-reducing effect than CNS metastases (3% vs. 2%), the latter are reported in more studies (17 vs. 11), increasing the power of the statistical test and allowing a statistically significant result (*p* = 0.014).

As already evidenced by previous reviews on real data [12,59], the results reported herein are consistent with data in the literature, as in published trials, the response rate was around 10 to 20% [5,6,7,40,55,61,62,63] and the median overall survival ranged from about 9 to 14 months [5,6,7,8,40,55,61,62,64].

### 5.2. Limitations

To the best of our knowledge this is the most recent meta-analysis on the efficacy of ICIs in NSCLC patients treated in second-line clinical practice.

The study has limitations inherent to the included studies. The real-world study design, heterogeneity of patients, lack of standardized treatment and the different collection processes for data make it advisable to be cautious when interpreting the results. The results of the meta-analysis were not corrected for a number of variables, such as type of NSCLC, PD-L1 expression levels or smoking status, which may have influenced the studies’ heterogeneity and the reliability of their results. In addition, the analysis did not differentiate between the types of ICIs or the combinations of drugs. Inclusion and exclusion criteria may also hamper the extension of the results to a broader range of NSCLC patients with chronic diseases.

Because of confounding, the studies we evaluated typically yielded low-quality evidence, according to the Cochrane risk of bias [65]. Nevertheless, since they were non-comparative clinical studies, two reviewers (R.A.F, P.T.) assessed the quality of the selected articles according to the study quality assessment tools for case-series studies [66]. An overall quality ranking was assigned to individual studies, graded as low, fair or good (Table S2). Out of the 32 studies entering the meta-analysis, 11 were judged good and 9 were graded as fair. The major concerns were the enrollment of non-consecutive patients and an adequate follow-up.

To counterbalance some limitations, we adjusted the pooled analysis for age and for clinical factors predictive of survival, such as ECOG PS and liver and CNS metastases.

## 6. Conclusions

The meta-analysis provided evidence of the benefits available to daily clinical practice with PD-1 and PD-L1 immunotherapy for previously treated patients with advanced squamous and non-squamous NSCLC. Despite the limitations due to the use of uncontrolled studies, the pooled data showed that the efficacy and safety findings in real-world practice were comparable to those in randomized clinical trials.

## Figures and Tables

**Table 1:** Characteristics of the real-world studies enrolled in qualitative synthesis and meta-analysis.

Author	N Patients	Age Median (Range)	Male %	Smoker %	Histology %	Stage %	PS %	Prior RT %	Metastases %	Cycles ICIs %	Line %
Adachi et al., 2020 [19]	296 n	70 (IQR: 64–76)	70	80	ADC: 62 Sq: 27 Other: 10	nr	0: 14 1: 61 2: 18 3: 5 4: 2	21	Brain: 26 Liver: 14	nr	2: 49 3: 23 4: 13 >4: 15
Chen et al., 2020 [20]	97 62 n 35 p	64 (IQR: 57–69) ≥65: 49	67	59	Non sq: 60 Sq: 40	III: 23 IV: 77	0–1: 84 ≥2: 15	28	nr	nr	2: 74 ≥3: 26
Dupont et al., 2020 [21]	191 n	63 (IQR: 56–68)	63	90	ADC:71 Sq: 23 Other: 6	III: 9 IV: 91	0: 19 1: 69 2: 8 3: 3 4: 0.5	nr	Brain: 23	nr	2: 48 3: 38 >3: 14
Figueiredo et al., 2020 [22]	219 n	64 (37–83) ≥75:15	70	69	Non sq: 88 Sq: 12	IIIB: 8 IV: 92	0: 12 1: 75 2: 13	nr	Brain: 0	14 (1–52)	2: 32 3: 38 ≥4: 29
Joris et al., 2020 [23]	324 n	65 (28–86) ≥70: 33 ≥75: 14	65	85	Non sq: 66 Sq: 28 Other: 6	nr	0–1: 69 ≥2: 27 Nos: 4	nr	nr	nr	2: 52 3: 28 >3: 20
Martin et al., 2020 [24]	109 n	Mean 65 (56–72)	58	75	Non sq: 78 Sq: 20 Nos: 2	IV:100	0–1: 83 2–3: 16 Nos: 2	61	nr	10 (IQR: 3–18)	Median before: 2 (1–4)
Pantano et al., 2020 [25]	294 n	67 (34–90)	68	86	ADC: 54 Sq: 42 Other:4	I-II: 11 * III: 23 IV: 65	0: 52 1: 44 2: 4	36	Brain: 14 Liver: 12	nr	2: 65 3: 23 >3: 12
Russo et al., 2020 [26]	187 n	67 (34–83)	73	90	Non sq: 54 Sq: 46	IIIB or IV	0: 45 1: 51 2: 4	nr	nr	nr	Median 2 (2–8)
Velcheti et al., 2020 [27]	349 p	68 (37–84) ≥75:25	57	92	Non sq: 58 Sq: 37 NSCLC nos: 4	IIIB-IV: 86	0: 26 1: 49 2: 18 3: 7	nr	Brain: 9	nr	2: 92 3: 7 ≥4: 1
Kim et al., 2020 [28]	83 n	60 (IQR: 53–68)	66	63	Non sq: 70 Sq: 30	IV:100	0–1: 76 ≥2: 24	nr	CNS: 34	nr	2: 43 ≥3: 57
Zhang et al., 2020 [29]	73 n	nr	75	nr	ADC: 57 Non sq: 37 Other: 5	III: 4 IV: 96	0–1: 90 ≥2: 10	nr	Brain: 44	nr	2: 82 ≥3: 19
Baldini et al., 2020 [30]	1959 n	66 (27–91)	68	73	Non sq: 81 Sq: 19	nr	0: 40 1: 52 2: 7	nr	Brain: 23 Liver: 20	7 (1–55)	2: 40 3: 29 ≥4: 30
Crinò et al., 2019 [31]	371 n	68 (31–91) ≥75: 19	80	83	Sq: 100	≥IIIB	0: 36 1: 58 2: 6	nr	CNS: 10 Liver: 17	Mean: 6 (1–22)	2: 44 3: 32 ≥4: 24
Fukui et al., 2019 [32]	52 n	69 (46–83) ≥75:21	71	82	ADC: 63 Sq: 31 Nos: 6	III: 25 IV:75	0: 37 1: 58 2: 4 3: 2	nr	Brain 15 Liver: 19	4 (1–43)	2: 42 3: 29 ≥4: 29
Grossi et al., 2019 [33]	1588 n	66 (27–89)	65	71	Non sq: 100	IIIb or IV	0: 41 1: 51 2: 7	nr	CNS: 26 Liver: 21	7 (1–55)	2: 24 3: 35 ≥4: 40
Lang et al., 2019 [34]	70 37 n 26 p 7 a	mean 66 (39–85)	60	80	ADC: 79 Sq: 30	III: 4 IV: 96	0: 24 1: 63 2: 13	nr	CNS: 23	nr	2: 100
Merino Almazan et al., 2019 [35]	221 n	Mean 64 >70: 27	84	69	Non sq: 38 Sq: 60	I-II: 8 * III: 38 IV: 54	0:28 1: 57 2: 14	9	Brain: 10 Liver: 19	Mean 9.7 (1–48)	2: 65 3: 28 ≥4: 7
Muchnik et al., 2019 [36]	75 65 n 6 p 4 other	Mean 74 (70–92)	52	nr	ADC: 68 Other: 32	IIIB: 3 IV: 97	0: 4 1: 47 2: 45 3: 4	nr	nr	nr	2: 16 3: 69 ≥4: 15
Weis et al., 2019 [37]	81 n	64	48	85	Non sq:60 Sq: 32 Other: 7	IV: 100	0: 17 1: 54 ≥2: 28	nr	nr	3 (1–18)	2: 64 ≥3: 36
	A 43	67	53	81	Non sq: 67 Sq: 28 Other: 5	IV:100	0: 16 1: 63 ≥: 21	nr	nr	4 (1–36)	2: 74 ≥3: 26
Schwartzberg et al., 2019 [38]	2071 1842 n 186 p 38 a	Mean 68 ≥75: 27	56	92	Non sq: 64 Sq: 31 Nos: 5	I-IIIA: 16 * IIIB-IV: 83 Nos: 2	0: 21 1: 50 2: 24 ≥3: 5	nr	nr	nr	2: 91 3: 9
El Karak et al., 2019 [39]	110 55 n 55 p	66	75	88	ADC: 57 Sq: 30 Nos: 13	IV: 100	Nr	nr	Brain: 17 Liver: 15	nr	2: 68 3: 24 ≥4: 8
Spigel et al., 2019 [40]	1426 n	67 (23–93) ≥70: 9	54	87	Non sq: 71 Sq: 28 Nos: 1	IIIB: 8 IV: 91	0: 23 1: 66 2: 9	nr	nr	nr	2: 39 3: 28 ≥4: 32
Areses Manrique et al., 2018 [41]	188 n	58 (45–81) ≥70: 7	77	91	ADC: 60 Sq: 35 Nos: 5	IIIB: 31 IV: 67	0: 8 1: 82 2: 10	nr	CNS: 22	6 (1–34)	2: 62 3: 24 ≥4: 14
Garde-Noguera et al., 2018 [42]	175 n	61.5 ≥70: 26.8	73	91	Non sq: 77 Sq: 23	III: 13 IV: 87	0–1: 81 2: 19	17	Brain: 22 Liver: 23		2: 37 3: 38 ≥4: 25
Fujimoto et al., 2018 [43]	613 n	Mean 66.9	71	79	ADC: 67 Sq: 22 Other: 10	IIIB: 6 IV: 94	0–1: 77 2: 15 3–4: 8	nr	nr	nr	2: 33 3: 25 ≥4: 42
Juergens et al., 2018 [44]	472 n	66 (36–92) >75: 13	43	54	Non sq: 73 Sq: 26 Other: 1	nr	0–1: 86 ≥2: 9 Nos: 5	nr	Brain: 13	nr	2: 44 3: 29 ≥4: 26
Kobayashi et al., 2018 [45]	142 n	67 (34–85) ≥75: 27	75	80	ADC: 58 Sq: 29 Other: 13	IIIA: 13 IIIB: 15 IV: 60	0: 30 1: 53 2: 11 3: 6	32	CNS: 19	nr	2: 40 ≥3: 60
Nakaya et al., 2018 [46]	101 n	69 (45–84)	77	84	Non sq: 63 Sq: 37	nr	0–1: 84 ≥2: 16	nr	nr	nr	2: 18 3: 28 ≥4: 55
Tamiya et al., 2018 [47]	201 n	68 (27–87)	67	78	ADC: 71 Sq: 21 Other: 8	IV:100	0: 16 1: 60 2: 16 ≥3: 7	nr	Brain: 25 Liver: 14	nr	≤3: 61 ≥4: 39
Toumoy et al., 2018 [48]	267 n	66 (41–86)	72	92	Non sq: 73 Sq: 27	III: 4 IV: 96	0: 16 1: 60 2: 24	52	Brain: 17 Liver: 21	6 (1–43)	2: 52 3: 33 ≥4: 16
Bagley et al., 2017 [49]	175 n	68 (33–88) ≥75: 25	46	84	Non sq: 76 Sq: 24	nr	0: 17 1: 58 2: 22 3: 3	nr	CNS: 31 Liver: 23	5 (1–24)	2: 54 3: 25 ≥4: 21
Brustugun et al., ** 2017 [50]	58 n	65 (32–88)	48	nr	ADC: 55 Sq: 41 Other: 3	I-II: 14 III: 42 IV: 62	0: 19 1: 57 2: 17 3: 7	nr	Brain: 0	8.5 (1–32)	2: 34 3: 47 ≥4:18
Kobayashi et al., 2017 [51]	50 n	65 (39–76)	60	62	Non sq: 88 Sq: 12	III: 20 IV: 58 rec: 22	0: 26 1: 64 2: 10	32	nr	4 (1–20)	2: 20 3: 18 ≥4: 62

* stage at diagnosis; ** not in meta-analysis; n = nivolumab; *p* = pembrolizumab; a = atezolizumab; nr = not reported; rec = recurrence after surgery.

**Table 2:** Outcome of the real-world studies enrolled in qualitative synthesis and meta-analysis.

Author	Follow-Up Months	Response % (95%CI)	PFS Months (95%CI)	OS Months (95%CI)	Adverse Event %
Adachi et al., 2020 [19]	26.6	ORR: 14 DCR: 55 CR: 1 PR: 13 SD: 40	3.0 (2.4–3.7)	10.5 (8.3–12.5)	nr
Chen et al., 2020 [20]	8.0	ORR: 17 DCR: 61 PR: 16 SD: 44	5.0	18	irAE any grade: 46 grade 3–4: 9
Dupont et al., 2020 [21]	24 (IQR: 20–29)	ORR: 26	2.8 (IQR: 1.6–10.4)	9.1 (IQR: 3.6–28.9)	any grade: 30 grade 3–4: 5
Figueiredo et al., 2020 [22]	17.1 (1–34.1)	ORR: 22 DCR: 53 CR: 1 PR: 21 SD: 31	4.9 (3.9–6.1)	13.2 (9.9–16.5)	any grade: 62
Joris et al., 2020 [23]	Nr	ORR: 21 CR: 4 PR: 17	3.8 (2.5–5.1)	8.6 (6.8–10.4)	any grade: 57 grade 3–4: 17
Martin et al., 2020 [24]	8.83 (0.2–33.7)	ORR: 29 DCR: 60 CR: 2 PR: 27 SD: 31	6.1 (range 2.4–13.1)	nr	any grade: 79 grade 2–3: 28
Pantano et al., 2020 [25]	nr	ORR: 34 DCR: 65 CR: 1 PR: 34 SD: 30	DSS: 14 (range 1-nr)	nr	any grade: 36 grade ≥ 3: 9
Russo et al., 2020 [26]	nr	ORR: 36 DCR: 67 PR: 36 SD: 31	7.0 (6–10)	13 (11–16)	nr
Velcheti et al., 2020 [27]	8.1 (0.01–39.2)	nr	nr	13.8 (11.0–16.5)	nr
Kim et al., 2020 [28]	nr	ORR: 7 DCR: 52 PR: 7 SD: 45	2.6 (0.8–4.3)	8.6 (5.6–11.6)	any grade: 32 grade 3–4: 8
Zhang et al., 2020 [29]	8	ORR: 22 DCR: 55 PR: 22 SD: 33	3.6	14.8	nr
Baldini et al., 2020 [30]	16.4	ORR: 18 DCR: 45 CR:1 PR: 17 SD: 27	3.3 (3.2–3.4)	10.3 (9.4–11.2)	irAE any grade: 7
Crinò et al., 2019 [31]	7.1 (0.1–16.4)	ORR: 18 DCR: 47 CR:1 PR:17 SD: 29	4.2 (3.4–5.0)	7.9 (6.2–9.6)	any grade: 29 grade 3–4: 6
Fukui et al., 2019 [32]	10.9 (IQ: 5.6–16.4)	ORR: 23 DCR: 44 PR: 23 SD: 21	2.1 (1.0–3.2)	1-year: 59.9	all any grade: 88 grade 3–4: 25 irAE any grade: 44 grade 3–4: 10
Grossi et al., 2019 [33]	8.1 (1–27.4)	ORR: 18 DCR: 44 CR: 1 PR: 18 SD: 26	3.0 (2.9–3.1)	11.3 (10.2–12.4)	any grade: 33 grade 3–4: 6
Lang et al., 2019 [34]	nr	ORR: 19 DCR: 37 CR: 1 PR: 17 SD: 19	4 (3–7)	18 (13–nr)	nr
Merino Almazan et al., 2019 [35]	nr	ORR: 17 DCR: 49 CR: 1 PR: 16 SD: 33	5.3 (3.2–7.3)	9.7 (7.6–11.8)	any grade: 71
Muchnik et al., 2019 [36]	nr	ORR: 36 DCR: 52 PR: 36 SD: 16	TTF 4.2 (2.0–5.8)	8.2 (4.8–13.6)	irAE any grade: 37 grade 3–4: 8
Weis et al., 2019 [37]	7.5 (0.5–35.4)	ORR: 15 DCR: 30 PR: 15 SD: 15	2.2 (1.7–2.8)	8.4 (6.3–11.2)	any grade: 70
	4.9 (0.6–13.5)	ORR: 14 DCR: 26 PR: 14 SD: 11	2.0 (1.8–2.7)	6.5 (4.7–nr)	any grade: 65
Schwartzberg et al., 2019 [38]	5.7 (0.03–32.5)	nr	nr	9.4 (8.9–9.9)	nr
El Karak et al., 2019 [39]	nr	ORR: 25 DCR: 57 CR: 3 PR: 22 SD: 31	4 (2.6–5.4)	8.1 (2.8–13.4)	irAE any grade: 18
Spigel et al., 2019 [40]	7.9	nr	nr	9.1 (8.3–10.4)	Total AE any gr:62 gr 3–4:15 irAE any gr:37 grade 3–4:6
Areses Manrique et al., 2018 [41]	nr	ORR: 25 DCR: 51 CR: 2 PR: 24 SD: 25	4.8 (3.7–6.0)	12.8 (9.1–16.6)	any grade: 78 grade 3–4: 5
Garde-Noguera et al., 2018 [42]	nr	ORR: 14	2.7 (2.0–3.4)	5.8 (3.7–7.9)	grade 3–4: 11
Fujimoto et al., 2018 [43]	nr	ORR: 20 DCR: 44 CR: 1 PR: 19 SD: 4	1-year: 18%	1-year: 54%	irAE grade ≥3: 11
Juergens et al., 2018 [44]	9.3 (0.03–24.5)	nr	3.5 (3.2–4.0)	12.0 (11–13.9)	Nr
Kobayashi et al., 2018 [45]	nr	ORR: 17 (12–24) DCR: 62 (54–70) PR: 17 SD: 45	1.9 (1.7–2.2)	nr	any grade: 45 grade 3–4: 13 grade 5: 1
Nakaya et al., 2018 [46]	8.9	ORR: 26 DCR: 61 PR: 25 SD: 35	3.2	17.0	all any grade: 60 irAE any grade: 40
Tamiya et al., 2018 [47]	12	ORR: 16 DCR: 52	2.9 (2.0–3.6)	nr	nr
Toumoy et al., 2018 [48]	nr	ORR: 23 DCR: 48 CR: 17 PR: 22 SD: 25	3.7 (2.9–4.5)	7.8 (6.3–9.3)	grade 3–4: 21
Bagley et al., 2017 [49]	nr	ORR: 19.4	2.1 (1.9–2.6)	6.5 (5.2–8.0)	irAE any grade: 16 grade 3–4: 3
Brustugun et al., 2017 [50] *	14	nr	4	12	any grade: 31 grade ≥3: 5
Kobayashi et al., 2017 [51]	nr	ORR: 18 DCR: 42 PR: 18 SD: 24	2.1	nr	nr

nr = not reported; irAE= immune-related adverse events; DSS = disease-specific survival; TTF = time to treatment failure; * not in meta-analysis.

**Table 3:** Estimated pooled ORR, DCR, progression-free survival (PFS) and overall survival (OS) by adjusting for age and CNS or liver metastases.

Outcome		Age	Liver Metastases		CNS Metastases	
	N		N		N	
ORR	22	0.21 (0.18–0.24)	12	0.20 (0.16–0.23)	19	0.20 (0.17–0.23)
DCR	18	0.52 (0.48–0.57)	10	0.47 (0.46–0.49)	16	0.51 (0.47–0.55)
PFS	18	3.25 (2.73–3.87)	11	3.11 (2.61–3.72)	17	3.15 (2.75–3.60)
OS	17	9.77 (8.53–11.20)	9	8.65 (7.23–10.36)	15	9.96 (8.43–11.76)

N = number of studies.

## Data Availability

Data sharing not applicable.

## Supplementary Materials

The following are available online at https://www.mdpi.com/2072-6694/13/6/1388/s1, Table S1: Role of performance status, age, CNS and liver metastases on studied endpoints evaluated in terms of odds ratio for ORR and DCR and of mean ratio for PFS and OS, Table S2: Risk of bias according to the Study Quality Assessment Tools for case-series studies.
