# Supplementary material for: Effectiveness and Safety of Immune Checkpoint Inhibitors for Patients with Advanced Non Small-Cell Lung Cancer in Real-World: Review and Meta-Analysis
Source: Cancers (Basel). 2021 Mar 19;13(6):1388. doi: 10.3390/cancers13061388 (PMC8003199; doi:10.3390/cancers13061388)
Supplement: Supplementary file 1 [file cancers-13-01388-s001.pdf]

Table 1S: Role of performance status, age, CNS and liver metastases on studied endpoints evaluated in terms of odds ratio for ORR and DCR and of mean ratio for PFS and OS

ORR	Odds Ratio		95% CI	P-value
Performance status	0.74		0.38-1.45	0.365
Age	1.02		0.96-1.08	0.576
Liver metastases (%)	0.96		0.91-1.02	0.215
CNS metastases (%)	0.99		0.97-1.01	0.230
DCR	Odds Ratio		95% CI	P-value
Performance status	0.65		0.34-1.26	0.190
Age	1.02		0.96-1.09	0.479
Liver metastases (%)	0.93		0.91-0.96	<0.001
CNS metastases (%)	1.00		0.98-1.01	0.758
PFS	Means Ratio		95% CI	P-value
Performance status	0.71		0.37-1.38	0.297
Age	0.98		0.92-1.04	0.400
Liver metastases (%)	0.97		0.92-1.03	0.297
CNS metastases (%)	0.98		0.96-0.99	0.014
OS	Means Ratio		95% CI	P-value
Performance status	0.75		0.45-1.26	0.262
Age	1.00		0.96-1.05	0.939
Liver metastases (%)	0.97		0.91-1.03	0.240
CNS metastases (%)	0.99		0.97-1.01	0.287

A unitary increase of the percent of patients with liver metastases reduced the occurrence of DCR by 7%, while the unitary increase of percent of patients with CNS metastases reduced median PFS by 2%

Table 2S: Risk of bias according to the Study Quality Assessment Tools for case-series studies [66]

Author	Was the study objective clearly stated?	Was the study population clearly described? *	Were the cases consecutive?	Was the intervention clearly described?**	Were the outcome measures clearly defined, valid, reliable?				Was the length of follow-up adequate?***	Were the statistical methods well-described?	Were the results well-described?	Quality Rating
					ORR	DCR	PFS	OS				
Adachi et al, 2020 (19)	yes	yes	yes	yes	yes	yes	yes	yes	yes	yes	yes	good
Chen et al, 2020 (20)	yes	no	yes	yes	yes	yes	no	no	yes	yes	yes	low
Dupont et al, 2020 (21)	yes	yes	yes	yes	yes	no	no	no	yes	yes	yes	good
Figueiredo et al, 2020 (22)	yes	yes	cd	yes	yes	yes	yes	yes	yes	yes	yes	good
Joris et al, 2020 (23)	yes	no	cd	yes	yes	no	yes	yes	cd	yes	yes	low
Martin et al, 2020 (24)	yes	no	cd	no	yes	yes	no	no	cd	yes	yes	low
Pantano et al, 2020 (25)	yes	yes	cd	yes	yes	yes	no	no	nr	yes	yes	fair
Russo et al, 2020 (26)	yes	no	cd	no	yes	yes	yes	yes	yes	yes	yes	low
Velcheti et al, 2020 (27)	yes	yes	cd	yes	no	no	no	yes	yes	yes	yes	good
Kim et al, 2020 (28)	yes	yes	yes	yes	yes	yes	yes	yes	cd	yes	yes	fair
Zhang et al, 2020 (29)	yes	no	cd	yes	yes	yes	no	no	yes	yes	yes	low
Baldini et al, 2020 (30)	yes	yes	yes	yes	yes	yes	yes	yes	yes	yes	yes	good

* yes if age, sex, histology, ECOG PS and presence of liver or brain metastases were reported; ** yes if ICIs lines were specified; *** yes if mean/median follow-up or overall survival ≥12 months; cd: cannot determine; nr: not reported

Table 2Sa: Risk of bias according to the Study Quality Assessment Tools for case-series studies [66]

Author	Was the study objective clearly stated?	Was the study population clearly described?*	Were the cases consecutive?	Was the intervention clearly described?**	Were the outcome measures clearly defined, valid, reliable?				Was the length of follow-up adequate?***	Were the statistical methods well-described?	Were the results well-described?	Quality Rating
					ORR	DCR	PFS	OS				
Crinò et al, 2019 (31)	yes	yes	cd	yes	yes	yes	yes	yes	cd	yes	yes	fair
Fukui et al, 2019 (32)	yes	yes	yes	yes	yes	yes	yes	no	yes	yes	yes	good
Grossi et al, 2019 (33)	yes	yes	cd	yes	yes	yes	yes	yes	yes	yes	yes	good
Lang et al, 2019 (34)	yes	yes	yes	yes	yes	yes	yes	yes	yes	yes	yes	good
Merino Almazan, et al, 2019 (35)	yes	yes	yes	yes	yes	yes	yes	yes	cd	yes	yes	fair
Muchnik et al, 2019 (36)	yes	no	yes	yes	yes	yes	yes	yes	cd	yes	yes	low
Weis et al, 2019 (37)	yes	no	cd	yes	yes	yes	yes	yes	cd	yes	**yes**	low
Schwartzberg et al, 2019 (38)	yes	no	cd	yes	yes	no	no	yes	cd	yes	yes	low
El Karak et al, 2019 (39)	yes	yes	cd	yes	yes	yes	yes	yes	cd	yes	yes	fair
Spigel et al, 2019 (40)	yes	no	cd	yes	no	no	no	yes	cd	yes	yes	low
Areses Manrique et al, 2018 (41)	yes	yes	cd	yes	yes	yes	yes	yes	yes	yes	yes	good
Garde-Noguera et al, 2018 (42)	yes	yes	cd	yes	yes	no	yes	yes	cd	yes	yes	fair

* yes if age, sex, histology, ECOG PS and presence of liver or brain metastases were reported; ** yes if ICIs lines were specified; *** yes if mean/median follow-up or overall survival ≥12 months; cd: cannot determine; nr: not reported

Table 2Sb: Risk of bias according to the Study Quality Assessment Tools for case-series studies [66]

Author	Was the study objective clearly stated?	Was the study population clearly described?*	Were the cases consecutive?	Was the intervention clearly described?**	Were the outcome measures clearly defined, valid, reliable?				Was the length of follow-up adequate?***	Were the statistical methods well-described?	Were the results well-described?	Quality Rating
					ORR	DCR	PFS	OS				
Fujimoto et al, 2018 (43)	yes	no	cd	yes	yes	yes	no	no	yes	yes	yes	low
Juergens et al, 2018 (44)	yes	yes	cd	yes	no	no	yes	yes	yes	yes	yes	good
Kobayashi et al, 2018 (45)	yes	yes	yes	yes	yes	yes	yes	no	nr	yes	yes	fair
Nakaya et al, 2018 (46)	yes	no	cd	yes	yes	yes	no	no	yes	yes	yes	low
Tamiya et al, 2018 (47)	yes	yes	yes	yes	yes	yes	yes	no	yes	yes	yes	good
Toumoy et al, 2018 (48)	yes	yes	yes	yes	yes	yes	yes	yes	cd	yes	yes	fair
Bagley et al, 2017 (49)	yes	yes	yes	yes	yes	no	yes	yes	cd	yes	yes	fair
Kobayashi et al, 2017 (51)	yes	no	cd	yes	yes	yes	no	no	nr	yes	yes	low

* yes if age, sex, histology, ECOG PS and presence of liver or brain metastases were reported; ** yes if ICIs lines were specified; *** yes if mean/median follow-up or overall survival ≥12 months; cd: cannot determine; nr: not reported
